# Thanking Cell & Bioscience reviewers

**DOI:** 10.1186/2045-3701-4-4

**Published:** 2014-01-14

**Authors:** Yun-Bo Shi

**Affiliations:** 1 Editor-in-Chief, Cell & Bioscience

## Abstract

**Contributing reviewers:**

The editors of *Cell & Bioscience* would like to thank all the reviewers who contributed to the journal in Volume 3 (2013).

## 

Daniel Buchholz

United States of America

Michael Buszczak

United States of America

Lu Cai

United States of America

Weili Cai

United States of America

Chia-Hsin Chan

United States of America

Wai Yee Chan

China

Chia-Hung Chen

Singapore

Dahua Chen

China

Jiandong Chen

United States of America

Shu-Hsia Chen

United States of America

Wanjun Chen

United States of America

Xin Chen

United States of America

Yu Chen

United States of America

Jocelyn Cote

Canada

Hongkui Deng

China

Xiao-Li Du

United States of America

Guoping Fan

United States of America

Zhaohui Feng

United States of America

Huichen Feng

United States of America

Burtram Fielding

South Africa

Chuanhai Fu

Hong Kong

Bin Gao

United States of America

Chan Gao

United States of America

Rui Gong

China

Nikolai Gorbunov

United States of America

Xiaoli Guo

United States of America

Georg Halder

Belgium

Kieran Harvey

Australia

Liqiang He

United States of America

Shanshan He

United States of America

Alexander Hergovich

United Kingdom

Wanjin Hong

Singapore

Peggy Hsieh

United States of America

Chuanshu Huang

United States of America

Jing Huang

United States of America

Li-Min Huang

Taiwan

Chien-Fu Hung

United States of America

Dong-Yan Jin

Hong Kong

Po-Ling Kuo

Taiwan

Min Gyu Lee

United States of America

Alex Li

China

Jun Li

China

Qiao Li

Canada

Shengwen Calvin Li

United States of America

Xiaofeng Li

United States of America

Hung-Yun Lin

Taiwan

Ying-Ju Lin

Taiwan

Junjun Liu

United States of America

Wansheng Liu

United States of America

Xiang Liu

United States of America

Jianfeng Lu

United States of America

Bingyu Mao

China

Yin-Yuan Mo

United States of America

Pawel Muranski

United States of America

Jian Pan

United States of America

Xiao Peng

United States of America

Robert Piper

United States of America

Rytis Prekeris

United States of America

Elena Susana Rivera

Argentina

Laurent Sachs

France

Chad Schwartz

United States of America

Ashwani Sharma

United States of America

Han-Ming Shen

Singapore

Yufang Shi

China

Jay Singh

United States of America

Steven Sperber

United States of America

Lishan Su

United States of America

Bing Sun

China

Sai-Wen Tang

United States of America

Michael Tsang

United States of America

Esther Verheyen

Canada

Lu Wang

United States of America

Sophia Wang

United States of America

Jen-Chywan Wang

United States of America

Chaochen Wang

United States of America

Lixin Wei

China

Yuehua Wei

United States of America

Tao Weitao

United States of America

Jiemin Wong

China

Patrick CY Woo

Hong Kong

Suh-Chin Wu

Taiwan

T-C Wu

United States of America

Jim Xiang

Canada

Gutian Xiao

United States of America

Wei Xu

United States of America

Hsin-Sheng Yang

United States of America

Xiaolong Yang

Canada

Yi-Yuan Yang

Taiwan

Venkat Yedavalli

United States of America

Paul Yen

Singapore

Yun Yen

United States of America

Zengqiang Yuan

China

Deliang Zhang

United States of America

Jiangwei Zhang

United States of America

Xiaohui Zhang

United States of America

Yanbin Zhang

United States of America

Bin Zhao

China

Richard Y Zhao

United States of America

Zhengyi Zhao

United States of America

Yong-Hui Zheng

United States of America

Weimin Zhong

United States of America

